# Identifying temporal patterns in trauma admissions: Informing resource allocation

**DOI:** 10.1371/journal.pone.0207766

**Published:** 2018-12-03

**Authors:** David P. Stonko, Bradley M. Dennis, Rachael A. Callcut, Richard D. Betzold, Michael C. Smith, Andrew J. Medvecz, Oscar D. Guillamondegui

**Affiliations:** 1 The Johns Hopkins Hospital, Department of Surgery, Baltimore, MD, United States of America; 2 Vanderbilt University Hospital, Division of Trauma and Surgical Critical Care, Nashville, TN, United States of America; 3 Department of Surgery, University of California, San Fransisco, San Fransisco, CA, United States of America; University of Kansas Medical Center, UNITED STATES

## Abstract

**Background:**

Increased knowledge of the temporal patterns in the distribution of trauma admissions could be beneficial to staffing and resource allocation efforts. However, little work has been done to understand how this distribution varies based on patient acuity, trauma mechanism or need for intervention. We hypothesize that temporal patterns exist in the distribution of trauma admissions, and that deep patterns exist when traumas are analyzed by their type and severity.

**Study design:**

We conducted a cross-sectional observational study of adult patient flow at a level one trauma center over three years, 7/1/2013–6/30/2016. Primary thermal injuries were excluded. Frequency analysis was performed for patients grouped by ED disposition and mechanism against timing of admission; in subgroup analysis additional exclusion criteria were imposed.

**Results:**

10,684 trauma contacts were analyzed. Trauma contacts were more frequent on Saturdays and Sundays than on weekdays (p<0.001). Peak arrival time was centered around evening shift change (6-7pm), but differed based on ED disposition: OR and ICU or Step-Down admissions (p = 0.0007), OR and floor admissions (p<0.0001), and ICU or Step-Down and floor admissions (p<0.0001). Step-Down and ICU arrival times (p = 0.42) were not different. Penetrating injuries peaked later than blunt (p<0.0001). Trauma varies throughout the year; we establish a high incidence trauma season (April to late October). Different mechanisms have varying dependence upon season; Motorcycle crashes (MCCs) have the greatest dependence.

**Conclusion:**

We identify new patterns in the temporal and seasonal variation of trauma and of specific mechanisms of injury, including the novel findings that 1) penetrating trauma tends to present at later times than blunt, and 2) critically ill patients requiring an OR tend to present later than those who are less acute and require an ICU or Step-Down unit. These patients present later than those who are admitted to the floor. Penetrating trauma patients arriving later than blunt may be the underlying reason why operative patients arrive later than other patients.

## Introduction

There has been significant research directed at understanding and streamlining patient flow, hospital organization and trauma systems in order to optimize patient outcomes [1–8] and efficiently utilize limited resources [9,4]. Furthermore, it is clear that understanding and describing patterns of trauma flow would be instrumental in allocating staff and resources across the trauma system. Despite the fact that scheduling and resource allocation decisions are based on “known” temporal patterns in trauma, literature describing these patterns is limited.

Prior work has detailed the effect that holidays [10], seasons, abrupt changes in weather [11–16], and other societal factors [10,15,17,18] have on trauma and emergency medicine epidemiology. These studies often set out to examine the role of how one or more factors are associated with or predict trauma volume. However, there has been no attempt to mathematically or graphically describe these patterns telescoping from hourly, weekly, seasonally and yearly patterns and across injury mechanisms and dispositions in one trauma center, a result that may prove useful for actually making adjustments to staffing or trauma system resources. Furthermore, there has also been no research describing how the temporal distribution of trauma varies with acuity.

The goal of this study is to characterize the temporal distribution of trauma admissions by analyzing and graphically representing how this distribution changes with different mechanisms of injury, types of trauma, and emergency department (ED) dispositions. We hypothesize that temporal patterns exist in the distribution of trauma admissionsand that these distributions differ depending on the traumatic mechanism, the type of trauma, and the level of care required. This knowledge and the easy to conceptualize and therefore utilize graphical depictions of these distributions will allow for evidence-based predictions of trauma volume, allocations of critical and often scarce resources, and staffing decisions.

## Methods

Data was acquired from the Trauma Registry of the American College of Surgeons (TRACS), a curated database including all admitted trauma patients in our level one trauma center from July 1, 2013 to June 30, 2016. The cohort included all adult trauma patients who presented to the emergency department during this period. Exclusion criteria were age less than 18 years old, primary thermal injury, or insufficient recording of emergency department admission or injury data. Emergency department (ED) arrival time was considered the admission time for each patient. Patients were examined by mechanism of injury, injury type (blunt versus penetrating), Injury Severity Score (ISS) [19], and ED discharge disposition: operating room (OR, any immediate transport to operation including interventional radiology), trauma ICU, trauma Step-Down unit, floor, or home. For subgroup analysis on ED disposition, patients who died in the ED were not included, nor were they included in this subgroup analysis.

We captured and plotted bivariate absolute and relative frequency data from all patients who met inclusion criteria in heat-maps showing time of day versus day of week. We then compared ED disposition by time of day for each mechanism of injury. For the overall cohort, we also examined admissions by ISS to evaluate the hourly admission prevalence for high (ISS≥15) and low (ISS<15) injury severity.

We plotted average daily admissions by presenting day of the year by tallying the total number of traumas. These data were normalized by the median number of traumas per day. LOWESS was used for fitting due to significant day-to-day variability in trauma volume. The median was chosen for normalization due to skewness in some trauma subtypes (e.g. the distribution of penetrating injuries/day was skewed to the right with rare days of high frequency). We performed this analysis for the cross section of all patients and on subsets of several mechanisms of injury. For the analysis of temporal patterns over the year we aimed to quantify the macro distribution of traumas for interpolating without goals of building a predictor model. Mathematical, statistical and graphical analysis was performed with offline MATLAB R2016b (9.1.0.441655) using Version 10.11.6. This study was conducted as approved by the Vanderbilt Institutional Reivew Board (IRB Number: 162071).

## Results

10,740 patients were identified. Of these, 56 patients were omitted due to missing a time or date stamp for entry to the Emergency Department (45) or for missing ISS score [11].(Table 1) Of the remaining 10,684 patients, 16 did not have an injury type of “penetrating” or “blunt,” and 78 did not have a recorded mechanism and were not included in subgroup analysis. This cohort was 74.9% male, mean age 45.9 (SD = 6.3), Table 1. Mean ISS was 14.7 (SD = 10.5, Median = 12, IQR = 11). 88.1% of traumas were blunt. 1.7% of patients died in the emergency department, and 15% required urgent or emergent transport from the ED to either the OR or interventional radiology.

**Table 1:** Patient cohort characteristics.

**Demographic and Injury Characteristics**		
Total Patients	10684	
Male	8005	74.5%
Mean Age	45.9	SD = 6.3
Mean ISS	14.7	SD = 10.5
Blunt	9415	88.1%
Penetrating	1253	11.7%
Unknown	16	0.1%
**Mechanism of Injury**		
Motorvehicle Collision (MVC)	3578	32.9%
Fall	3302	30.4%
Motorcycle Collision (MCC)	842	7.8%
Gun	764	7.0%
All-terrain Vehicle (ATV)	46	0.4%
Other	2254	20.7%
Unknown	78	0.7%
**Emergency Department Disposition**		
Operating Room	1622	15%
Trauma ICU	3118	28.7%
Trauma Step-Down	4075	37.5%
Floor Room	1546	14.3%
ED Death	187	1.7%
Home	314	2.9%

Using a heat map, the times of the day and week which have the fewest trauma activations (indicated by the blue colors of the heat map) are between 05:00–10:00, Fig 1. Our findings also demonstrate that there is day-to-day variation. Trauma activations are most common in the weekday evening hours and have multiple peaks on weekend evenings. Qualitatively, on most weekdays, the average nadir of trauma contacts occurs between 05:30 and 06:30, and the frequency of trauma contacts increases throughout the day, peaking between 18:30 and 19:30 Monday through Thursday. On Saturdays and Sundays, the morning nadir occurs at the 07:00 and early 08:00 hours, respectively. The evening peak also occurs later on Friday and Sunday evening compared to other weekdays. Additionally, there are more trauma contacts on weekend days than on weekdays (p<0.001).

**Fig 1:**
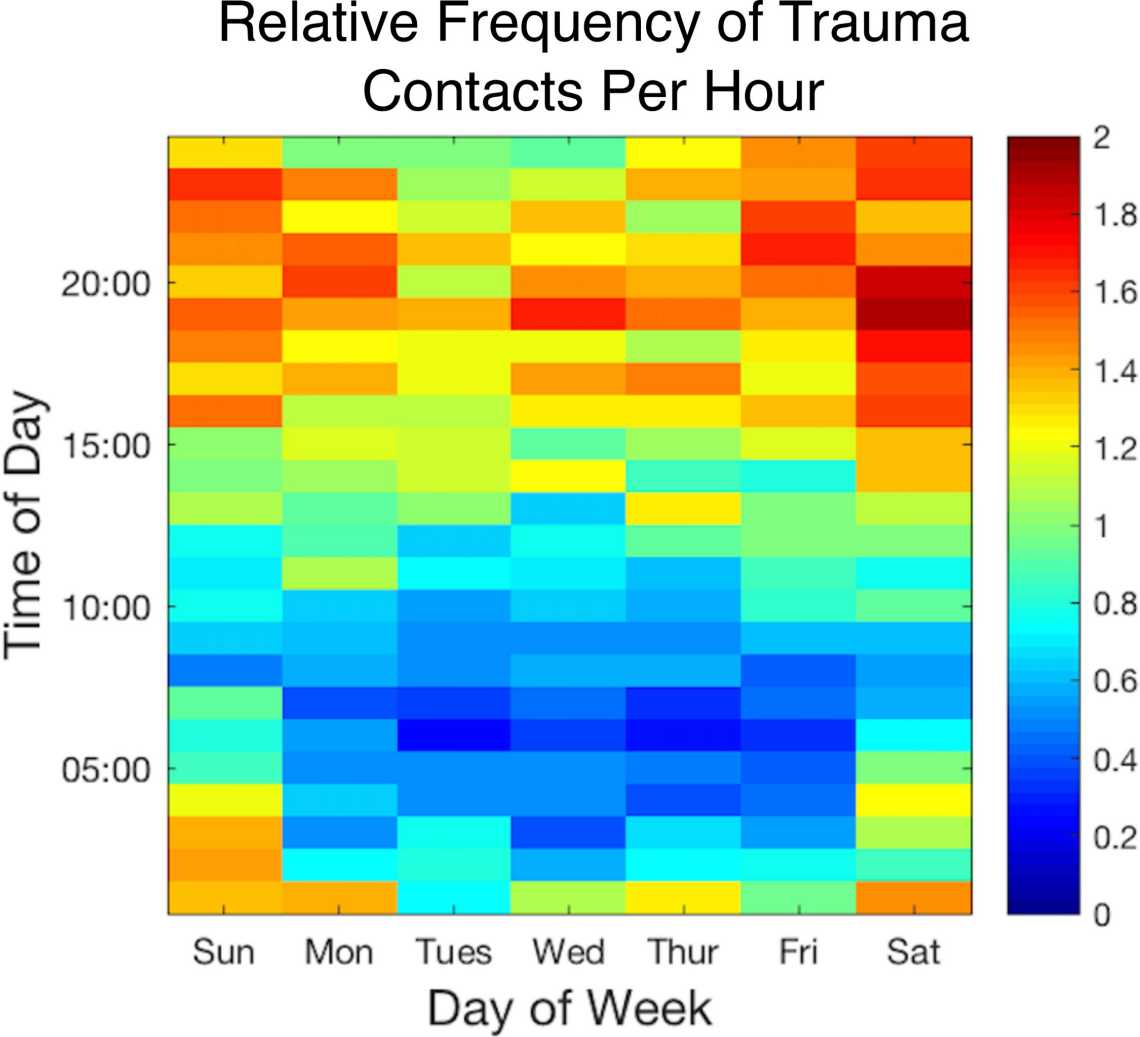
Heatmap of trauma admissions by time of day versus day of week. Each block of the heatmap represents a one hour block of one day of the week over the three year study period. The color corresponds to the relative frequency of contacts per hour as described by the color bar to the right of the image, where 1 represents the mean number of trauma contacts per hour. Weekend days have more trauma than weekdays, P<0.001. Mornings (4AM– 8AM) have less trauma than evenings (4PM– 8PM) on all days of the week, P<0.001.

**Fig 2:**
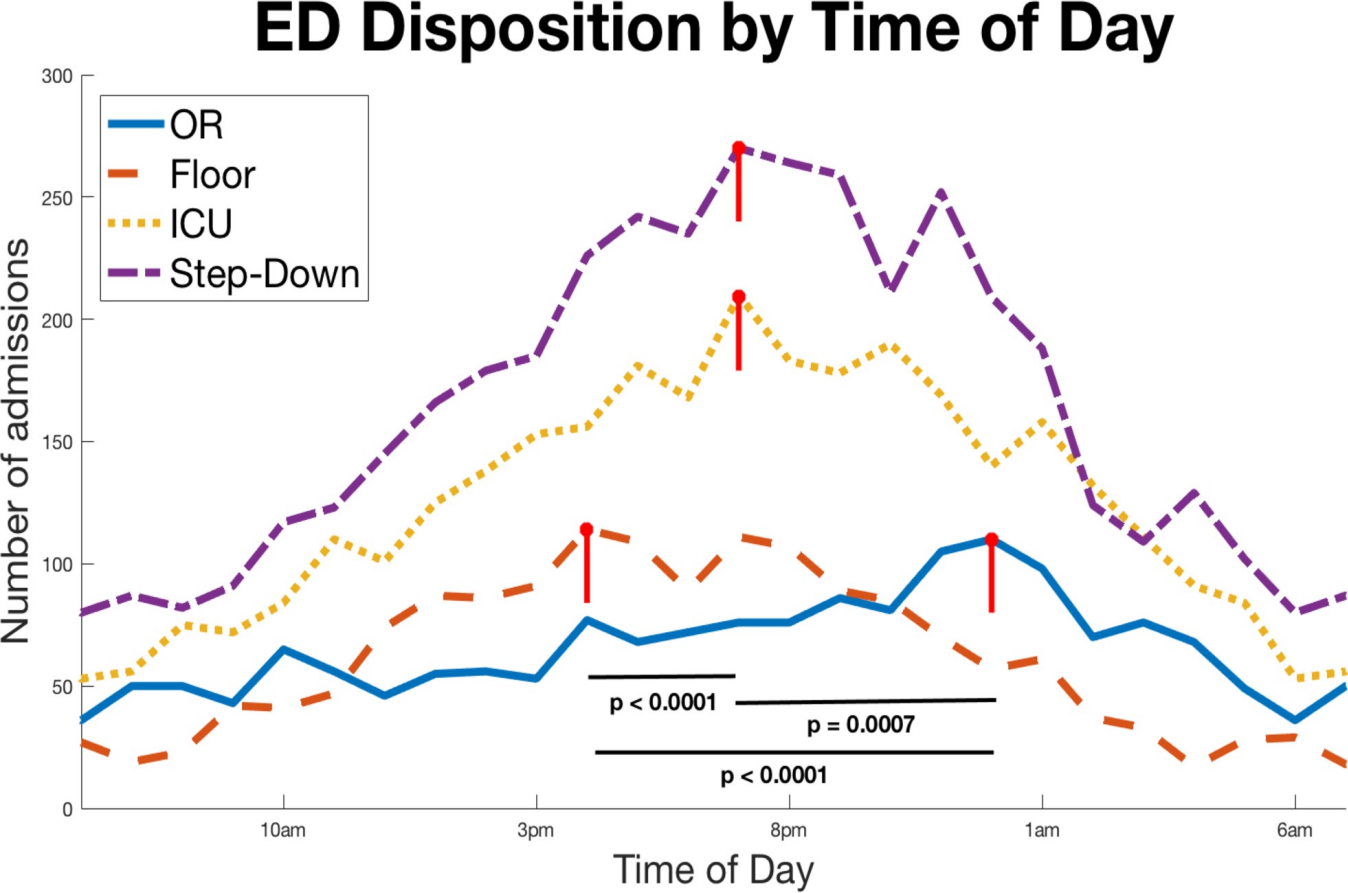
Absolute frequency of trauma patients sent from the emergency department to each of these disposition by time of day. Patients admitted to the floor peak earliest (red-dashed). Step-down (purple-dashed) and ICU (yellow-dashed) peak next during the day, and operative patients present latest.

Fig 2 shows the ED disposition data for all trauma contacts as the absolute frequency versus the time of day for each of the ED dispositions of interest. These data demonstrate that admissions vary throughout the day but that each partition of the cohort has a different peak utilization: those admitted immediately to the floor peak before ED admission to the ICU, Step-Down and OR patients. Patients that leave the ED to Step-down and ICU rooms peak next, and patients who required immediate OR present latest in the shift, typically near midnight. The time of admission for the OR shows less temporal variation over the day with the relative peak time of admission occurring at the 23:00 to 24:00 hour. In contrast, the peak admission time for patients in need of an ICU or Step-Down unit occurs earlier than those in need of immediate operation (p = 0.0007), and the peak admission time for patients in need of a floor bed from the emergency department occurs even earlier than patients who leave the ED to the ICU and Step-Down units (p<0.0001).

**Fig 3:**
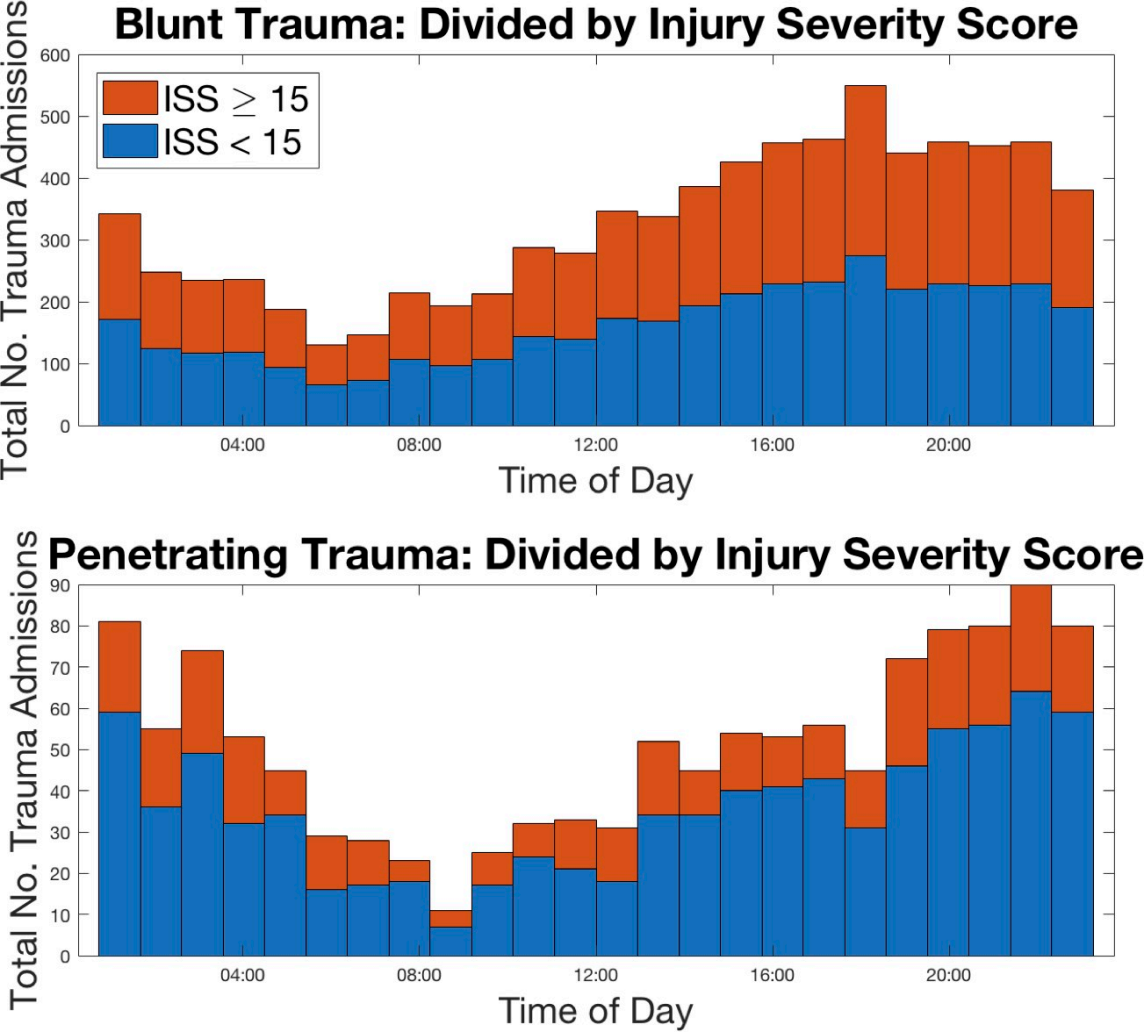
Absolute frequency of trauma admissions versus time of day partitioned by blunt and penetrating and divided by injury severity score.

When temporal patterns were considered by mechanism, there was variation throughout the day for both blunt and penetrating mechanisms, Fig 3; however, the pattern was different. Blunt trauma was lowest at the 05:00 hour and then had a steady rise throughout the day, peaking at the 20:00 hour. Penetrating trauma has its trough later than blunt trauma: at the 08:00 to 09:00 hour and then peaks later than blunt trauma, at the 22:00–23:00 hour and remaining elevated into the early morning hours.

**Fig 4:**
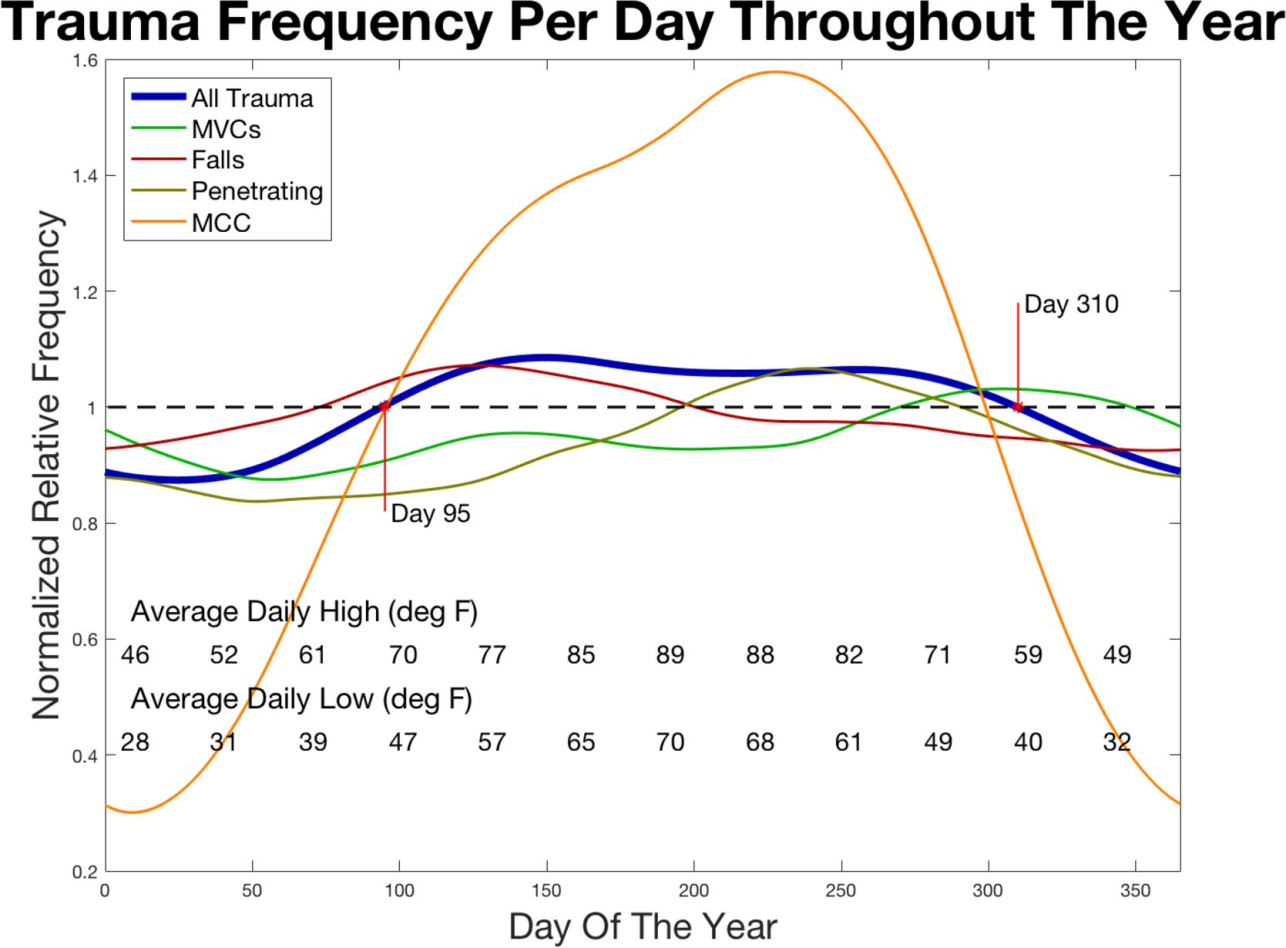
Smoothed, normalized relative trauma frequency per day throughout the year. All trauma (blue) shows above-median trauma during mid year. Several of mechanisms of injury (see legend) are considered additionally. The first point marks the date (day 95: April 5^th^) that this institution tends to see its median number of traumas. The second point (day 310: November 6^th^) denotes the end of the above-median yearly peak in trauma contacts per day.

We also considered the trauma distribution over the year; the normalized median frequency of all traumas versus day of the year is shown in Fig 4. The blue line represents the smoothed moving-median of traumas throughout the year. Trauma frequency peaks mid-year. The incidence of all trauma (blue line) tends to be highest in summer, increasing throughout spring and becoming less frequent in the fall, representing a “trauma season” with an 8% above-median rate of trauma per day in mid-summer compared to 11% below average number patients admitted per day in January; a nearly 20% swing from summer to winter. Although there is a clear peak in the summer months, there is seasonal variation by mechanism. MCCs show the greatest dependence on seasonality with the vast majority occurring during summer with less than 70% below-median number of MCC trauma per hour in winter, compared to nearly 60% above-median in mid-summer. Falls tend to be slightly more common in spring while motor vehicle crashes(MVCs) tend to be more common in fall yet demonstrate less dramatic seasonal dependence as MCCs. Similarly, penetrating mechanisms of injury show less dramatic seasonal dependence when compared to motrocycle-related trauma but still do suggest that there is some dependence on time of year.

## Discussion

We examine the temporal distribution of trauma at the hourly level with subgroup analysis into mechanism and ED disposition to identify unique patterns that relate the severity of trauma to timing of presentation. We also assess weekly and seasonal variation in an easy-to-understand graphical analysis approach. This complements literature highlighting the statistics associated with specific points within this distubution but may be more useful in clinical and educational practices. Specifically, this information may indicate the most effective time for handoffs and staffing changes by predicting interruptions, which have been shown to be detrimental to patient outcomes and to increase resource utilization [20–22]. Residents and other trainees may benefit by predicting times of high volume for those interested in gaining more clinical experience and lulls in trauma activations to allow for uninterrupted education and didactic activities. For example, Fig 1 could be interpreted by nursing leadership estimating how many beds in the ICU may fill during busy patient flow over the upcoming weekend or by a residency program director scheduling lectures that do not overlap with times of high-volume trauma activations.

We show that the daily nadir of trauma occurs between 05:30 and 06:30 on weekdays and between 07:15 and 08:15 on the weekends, and the frequency of traumas increases throughout the day. The peak tends to occur between 18:30 to 19:30 Monday through Thursday. This finding is particularly important as most evening handoffs of nursing, resident, and attending providers tend to occur simulatenous with the peak of activations. The period between Friday evening and Monday morning has a distinctly different pattern, where the morning nadir occurs later, at 07:00 to 09:00 and the evening peak moves later and lasts longer. This could be the product of increased and delayed evening activity associated with weekends in an urban area [23,24].

This information is efficacious in understanding the additional stress and burden placed on often over-taxed systems of care when we attempt transitions during peak resource needs. The ability to use this data to re-engineer our workflows could be transformative for healthcare needs as we balance demand and supply. This may translate into evening rounds occurring earlier or later, leveraging the predictable circadian cycle of trauma activations.

Blunt trauma comprises the vast majority of all trauma at this and most other centers [25–27] and consequently drives the patterns of overall trauma admissions. However, during subgroup analysis, we observe a relative delay in timing of penetrating traumas. These reach their trough just before 09:00 and peak at 22:00–23:00, both of which are much later than their blunt counterparts. This same pattern is seen in patients who transfer from the emergency department directly to the OR. These tend to arrive later than those who transfer elsewhere in the institution. This pattern is most likely due to late penetrating trauma and is driving OR usage compared to other dispositions.

Higher acuity and resource-demanding dispositions occur later in the evening (immediate OR > ICU and Step-Down > floor). This is important because it significantly influences the needs of the hospital workforce and resource utilization. A trauma OR activation requires a trauma surgeon, surgical assistant, an anesthesia team, and numerous operating room staff to mobilize often at a moment’s notice. It is not a simple increase in the amount of resources being dedicated to the patient as a floor admission may be. This pattern indicates that penetrating traumas and the specific needs required should be considered distinct from a resource allocation perspective. Perhaps healthcare systems with high trauma volume may choose a modified night staffing model that reflects the arrival of high acuity patients, as opposed to standard practiceswhich have the least redundant workforce at night.

We define a period of the year when expectations of above median trauma volume occur, and for the region studied this appears to occur from early April to early November, Fig 4. One limitation of this analysis is that it utilized smoothing to minimize the effect of specific events throughout the year, such as holidays or days with very atypical weather patterns which are known to be important. While this allowed us to obtain a better understanding of the overall trends of trauma throughout the year, it prevents us from analyzing the effect that one such event might have, though this is already described in the literature and, therefore, was not a goal of this analysis [10–16].

Subgroup analysis of this same data also provides interesting conclusions. One may expect falls to be more prevalent in winter because of icy conditions, but we found that they have a proponderance for occurring in spring. However, icy conditions occur infrequently in the study region, averaging only 10.7 inches of snow and ice per year [28]. Falls caused by snow and ice were too rare to analyze. MCCs had the greatest seasonal variation, seen far more commonly in the warm weather months. They were over twice as common Saturday and Sunday as compared to weekdays. This distribution is likely due to recreational motorcycle use in warmer weather months and on weekends. Automobile utilization occurred more consistently throughout the year for daily commutes and subsequently had far less seasonal variation.

A limitation of this study is the use of a single institution with one climate. This is the only level 1 trauma center in region that services 50,000 square miles and includes both rural and urban areas. In warmer climates with less seasonal variation in weather, one would expect differing patterns of peaks in the year, but that the relative day to day variation would remain consistent. Different trends may appear in centers of other size, locations, volume or mechanism of injury ratios. This study’s extension may also be limited in application to pure urban or rural trauma center, those with different ratios of blunt and penetrating trauma, or urban trauma systems that share patients among multiple trauma centers within close proximity. Therefore, while this study reflects the particular nuances of the geographic and trauma demographics of the southeast, other institutions or regions may still consider using this methodology to evaluate the unique circumstances of their trauma population. An additional limitation to the study is that the cross-sectional study design creates the potential for bias given unmeasured confounders, including the diversion status of other area hospitals.

## Conclusion

We hypothesized that there were temporal patterns in the distribution of trauma admissions. Using novel graphical and numeric representation of the distribution of trauma in time, we show that weekends have higher trauma volume than weekdays, evenings have more trauma than mornings, and trauma occurs later on weekends than on weekdays. We also demonstrate that penetrating trauma admissions and operating room activations occur later than other trauma, that higher acuity ED dispositions tend to be utilized later than less acute dispositions, and that this may be driven by the temporal relationship of penetrating versus blunt trauma. On average, trauma volume peaks during mid-year with the trauma season extending from early April to early November, and seasonal variation is associated with differential prevalence of some trauma, such as falls and MCCs. Understanding these patterns is immediately useful for staff and trainee scheduling, and it allows for accurate predictions of high or low trauma volume periods.

## Supporting information

S1 File Dataset. (ZIP)
